# Targeting Cardiovascular Diseases by Flavonols: An Update

**DOI:** 10.3390/nu14071439

**Published:** 2022-03-30

**Authors:** Aleksandra Kozłowska, Dorota Szostak-Węgierek

**Affiliations:** 1 Department of Social Medicine and Public Health, Medical University of Warsaw, Oczki Str. 3, 02-007 Warsaw, Poland; aleksandra.kozlowska@wum.edu.pl; 2 Department of Clinical Dietetics, Faculty of Health Sciences, Medical University of Warsaw, E Ciołka Str. 27, 01-445 Warsaw, Poland

**Keywords:** flavonols, quercetin, cardiovascular diseases, inflammation

## Abstract

Flavonols are one of the most plentiful flavonoid subclasses found in natural products and are extensively used as dietary supplements. Numerous in vitro and in vivo studies have shown the cardioprotective properties of flavonols, especially quercetin. This group of substances exerts positive impacts primarily due to their antiatherogenic, antithrombotic, and antioxidant activities. The potential of flavonols to promote vasodilation and regulation of apoptotic processes in the endothelium are other beneficial effects on the cardiovascular system. Despite promising experimental findings, randomized controlled trials and meta-analyses have yielded inconsistent results on the influence of these substances on human cardiovascular parameters. Thus, this review aims to summarize the most recent clinical data on the intake of these substances and their effects on the cardiovascular system. The present study will help clinicians and other healthcare workers understand the value of flavonol supplementation in both subjects at risk for cardiovascular disease and patients with cardiovascular diseases.

## 1. Introduction

Cardiovascular diseases (CVDs) are a group of heart and blood-vessel disorders. They include coronary heart disease (CHD), cerebrovascular disease, peripheral arterial disease, rheumatic heart disease, congenital heart disease, deep-vein thrombosis, and pulmonary embolism. CVDs, particularly ischemic heart disease (IHD) and stroke, are the major cause of global mortality and prolonged disability [1]. Extensive epidemiological studies have shown that principally modifiable risk factors (e.g., high blood glucose, hypertension, dyslipidemia, and obesity), as well as lifestyle features (e.g., unhealthy dietary patterns, low physical activity, and alcohol and tobacco use), are considered as important risk factors for CVDs [2,3,4,5]. Thus, public health experts advocate for medication use, lifestyle changes, and dietary modifications for preventing the incidence of CVDs and their complications, especially among patients with, or at high risk of, CVDs [6,7].

Reduced risk of chronic diseases, including CVDs, has been linked to high consumption of fruits and vegetables [6,8,9]. Besides their vitamin and fiber content, these foodstuffs are known for high levels of flavonoids in general, and more specifically of flavonols. Among the flavonols, the five most abundant in plants are kaempferol, quercetin, fisetin, isorhamnetin, and myricetin [10]. Flavonols, and especially quercetin, show a broad range of biological functions. As a result of their action on cell-signaling pathways associated with oxidative stress and inflammation, they may improve lipid metabolism, vascular function, blood pressure, and glucose metabolism [11,12,13]. Based on the role of these effects in the pathogenesis of CVDs, applications of flavonols have been considered to decrease or prevent the progression of CVDs.

In recent years, researchers have been debating whether discoveries made in vitro and in vivo with quercetin and other flavonols have any relevance in human clinical trials. The scientific community has established a great interest in the characterization and validation of flavonoids in CVDs by exploring their molecular processes and conducting dozens of new clinical trials.

In the query performed in the ClinicalTrials.gov database in January 2022, only the keyword “quercetin” identified 101 clinical studies in which this flavonol was registered. Thus, the present study aims to update and provide a more comprehensive estimate of the association between flavonol intake, either as dietary supplements or pure compounds, flavonoid mixtures or extracts, and their cardioprotective effects in humans.

## 2. Materials and Methods

To assess the effects of flavonols on cardiovascular health, the Medline (http://www.ncbi.nlm.nih.gov/pubmed) was searched using a combination of the following queries in titles and abstracts:

(flavonols OR kaempferol OR quercetin OR fisetin OR isorhamnetin OR myricetin OR rutin) AND (“cardiovascular disease” OR ”heart diseases” OR stroke OR blood pressure OR hypertension OR hyperlipidemia OR cholesterol OR triglycerides OR obesity OR “blood glucose” OR “endothelial function” OR atherosclerosis OR “coronary heart disease” OR “cerebrovascular disease” OR “peripheral arterial disease” OR “rheumatic heart disease” OR “congenital heart disease” OR “deep vein thrombosis” OR “pulmonary embolism” OR “ischemic heart disease”).

The search was limited to clinical trials, meta-analyses, or randomized controlled trials. The literature was searched from 1 January 2017 to 8 January 2022. As a result, we retrieved 19 clinical papers for full-text reading. It is worth noting that the majority of the studies were performed with quercetin. The available data indicate scare numbers of clinical trials of kaempferol, isorhamnetin and tamarixetin, fisetin, and myricetin.

We did not introduce any time restrictions on the publication of preclinical data. However, we provided an update of the most recent articles (published in 2021) that may be not known to the readers.

## 3. Flavonols and Their Metabolites

Flavonoids are a large group of polyphenols found in plants. They may be further split into six subclasses: anthocyanins, flavanols, flavanones, flavones, flavonols, and isoflavones. Subgroups of flavanonols, chalcone, and neoflavonoids have also been distinguished by some authors [10,14,15]. Flavonols (together with flavanols) are the most ubiquitous flavonoids found throughout the plant kingdom. Kaempferol, quercetin, fisetin, isorhamnetin, and myricetin are the most common dietary flavonols.

**Figure 1:**
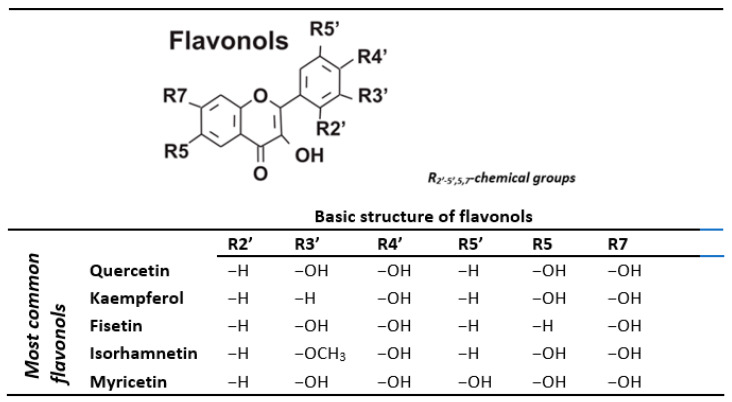
Chemical structure of the main flavonols present in plant food, based on [17,19].

All naturally occurring flavonoids are composed of three rings (Figure 1) and are subdivided into various subclasses depending on the degree of unsaturation of the central ring and the number of its carbon to which the peripheral ring is attached. Flavonoids are found in the form of aglycones, glycosides, and methylated derivatives [16]. The free forms of flavonols are aglycones, which have lipophilic properties. Because aglycones are unstable, quite reactive, and have low solubility, flavanols possess a tremendous diversity of glycoside forms, both in terms of the position and type of sugar moieties. The sugar substituents most frequently attached to flavonols are monosaccharides, such as glucose, rhamnose, galactose, arabinose, xylose, and the disaccharide rutinose, which is composed of glucose and rhamnose connected by a β-glycosidic bond [17]. Glycosylation enables the preservation of the hydrophilicity and stability of hydrophobic flavonoids. Further, the conjugation of flavonoid aglycones and glycosyl groups can alter flavonoids’ biological activity, reduce toxic and side effects, and enhance specific targeting [18].

Figure 1 presents the basic structure of flavonols and examples of specific substances in the flavonols subclass.

## 4. Food Sources and Dietary Intake

Flavonols are found in various foods of plant origin. Table 1 shows their main dietary sources, which include capers, parsley, elderberry juice, and sorrel [20]. It should be noted that the presence of particular flavonols in vegetables and fruits may vary significantly depending on the cultivar, climate (sun exposure, precipitation), seasonality, species of plant, food processing, and storage conditions [21,22]. Differences in flavonols’ contents between samples of the same plant species are usually moderate [20]. The method of cooking food also has an impact on flavonol content. A decrease of 50–60% in quercetin was recorded in red and yellow onion boiled for 60 min. However, quercetin derivatives were transferred into the liquid part of the soup [23,24]. Similarly, boiling led to a considerable loss of quercetin and kaempferol in broccoli [25].

The amount of flavonol intake from food is primarily determined by individual dietary habits. Quercetin, one of the most abundant flavonols, is consumed in the largest quantities (about 14 mg per day in Europe) [26]. According to new research, the average daily intake of flavonols differs by country. In Europe, the estimated daily intake was on average 18 mg/day, while in the United States (US) population and among Japanese adults, mean intakes were 18 and 58 mg/day, respectively [26,27]. Similarly to US adults, habitual dietary intake of this flavonoid subclass in a cohort of United Kingdom women was on average 58.3 mg/day, and the major food sources included onion and spinach [28]. However, a comparison of the results of nutritional assessments of flavanol intake is based on diverse data sources which may differ and be imprecise. The most commonly used databases of flavonol content in foods are the US Department of Agriculture (USDA) databases (https://fdc.nal.usda.gov/) and the online Phenol-Explorer database (https://phenol-explorer.eu). Because only a limited number of foods have been investigated for polyphenol content using various analytical methods, these tables may be of limited utility. It is interesting to note that the improved availability of food-composition data for flavonoids has enabled a more precise measurement of intakes [29].

## 5. Bioavailability

The structural class, the total amount of hydroxyl groups, replacement of functional groups around their nuclear structure, and degree of polymerization all influence flavonol bioavailability, metabolism, and biological activity [17,30]. Estimating the bioavailability of flavonols, which is defined as the portion of an initially administered dose that reaches the systemic circulation unchanged after a single oral dose, is essential in determining the potential mechanisms of its action [31]. It is well-known that flavonols are poorly absorbed, with an extremely low oral bioavailability [17,32,33]. The investigation on the pharmacokinetics of quercetin, the well-studied flavonol, in humans suggested very poor oral bioavailability of this substance (2–4%), mostly due to its extensive metabolism and/or rapid elimination. However, conjugated forms, the naturally occurring form of quercetin, have higher bioavailability than the free forms, and the estimated absorption ranges from 3% to 17% in healthy individuals [34]. The features of linked sugar moieties and their solubility in water or lipids are the factors that most impact and normally improve quercetin bioavailability [31]. Thus, the present research is focused on various novel formulation strategies such as lipid vesicles, polymeric nanoparticles, solid-lipid nanoparticles, complexation techniques, liposomes, and micelles, which appear to increase flavonol solubility and bioavailability [33,35,36].

According to literature data, flavonols are rapidly enzymatically hydrolyzed and absorbed in the intestine, where they undergo glucuronidation and sulfidation by phase II enzymes in epithelial cells and in the liver. After metabolization, these substances are distributed throughout the whole organism, where they are transported to target tissues. Finally, flavonols and their metabolites are excreted through feces, urine, and exhalation of carbon dioxide [17]. However, flavonols metabolism in body tissues is not well-understood. Evidence suggests that intestinal bacteria are deeply involved in the production of flavonol metabolites [37,38]. Furthermore, the action of these compounds varies across individuals based on food matrix and processing; then, enzymatic levels are determined by genetic variables and diet, age, and sex, and finally microbial functionality.

## 6. Flavonols and Their Cardioprotective Activity

Flavonols have numerous cardioprotective properties, such as platelet stabilization, anti-inflammatory, antidiabetic, antihypertensive, and hypocholesterolemic activity [39,40,41,42,43,44,45,46,47,48,49,50]. To the best of our knowledge, quercetin is the most widely distributed and well-known flavonol obtained from natural sources, as well as the best-studied flavonol, in both in vitro and in vivo studies [51]. Quercetin can protect the cardiovascular system by multiple pathways.

Lastly, more in-depth research revealed that quercetin’s positive effects on myocardial inflammation were linked to its antioxidant and anti-inflammatory activity. Chang et al. showed that quercetin pretreatment protects human cardiomyocytes from hypoxia-induced oxidative-stress damage by inhibiting reactive-oxygen-species (ROS) production and oxidative-stress damage, improving mitophagy and energy metabolism, regulating mitochondrial/endoplasmic-reticulum function, and reducing apoptosis [52]. Furthermore, Cheng et al. studied the effects of quercetin on mouse models of myocardial fibrosis and heart failure, finding that quercetin administration reduced myocardial fibrosis and enhanced cardiac function in high-glucose-exposed inflammation-injury cardiomyocyte cell line HL-1 [53]. These effects were partially mediated by increased mitochondrial energy metabolism as well as regulation of mitochondrial fusion/fission and mitochondrial biosynthesis. In addition, quercetin reduced the incidence of heart failure by inhibiting the inflammatory response and oxidative-stress injury, protecting mouse cardiomyocytes under inflammatory conditions, and improving myocardial fibrosis [53]. Similarly, in the ischemia/reperfusion-induced rat model, quercetin exerted cardioprotective effects by inhibiting inflammatory responses and improving contractility potential. Quercetin was associated with diminished levels of interleukin 1β (IL-1β), IL-6, and tumor necrosis factor-alpha (TNF-α). Further, quercetin’s anti-inflammatory effects might be mediated in part by mitochondrial K-ATP (mitoKATP) channels and the nitric-oxide (NO) system [54].

In animal and cell-culture studies, flavonols, particularly quercetin, were observed to have antihypertensive effects. The angiotensin-converting enzyme (ACE) is well-known for its role in the renin-angiotensin-aldosterone system (RAAS), which regulates plasma sodium concentration, arterial blood pressure, and extracellular volume [55,56]. Inhibitors of ACE are widely used for the treatment of hypertension. Interestingly, in vitro study showed that the flavonol-rich extract of *Actinidia macrosperma* (a wild kiwifruit) inhibited ACE [57]. These findings demonstrated that flavonol may inhibit enzyme activity by nonspecific binding to the enzyme or by competing for the active side with the substrate. Findings from this in vitro study indicate that flavonols may have the potential to exert an antihypertensive effect in vivo. Galindo et al. evaluated the effects of quercetin administered orally on different cardiovascular protective effects in spontaneously hypertensive rats (SHR) [58]. Intake of quercetin reduced the systolic blood pressure, normalized the heart rate, and reduced heart hypertrophy. Further, quercetin allowed aortic relaxation in SHR by enhancing NO and decreasing the expression of NADPH oxidase in some subunits. Similarly, Elbarbry et al. determined the effect of oral administration of quercetin in drinking water (10, 30, and 60 mg/L) on blood pressure and arachidonic acid (AA) metabolism in spontaneously hypertensive rats [59]. Medium- and high-dose quercetin ameliorated blood pressure in SHR. Further, by reducing the activity of the key enzymes involved in AA metabolism (cytochrome P450 4A and soluble epoxide hydrolase, sEH), quercetin influenced AA metabolism in the kidney [59]. Thus, the antihypertensive effects of quercetin may be partially due to its effect on AA metabolism, although the mechanism underlying the improvements in blood pressure by this substance is still unknown. The synergic effect of quercetin metabolites on blood pressure has been also investigated. Najmanová et al. showed that combinations of three colonic metabolites of quercetin, 3,4-dihydroxyphenylacetic acid (DHPA), 4-methylcatechol (4MC), and 3-(3-hydroxyphenyl)propionic acid (3HPPA) had a more pronounced antihypertensive effect than single metabolites in SHR [60]. The longest-lasting effect was achieved by combining 3HPPA and 4MC.

Thrombosis, myocardial infarction, stroke, and atherosclerosis in coronary or carotid arteries are caused by dysregulation of blood-platelet activation [61]. The cardioprotective function of flavonols is greatly influenced by its antiplatelet-aggregation effects via a variety of mechanisms, the most significant of which seems to be suppression of the arachidonic acid-based pathway. The *Campomanesia adamantium* peel extract, rich in quercetin and myricetin, exerted antiplatelet activity in the platelet aggregation caused by arachidonic acid (AA) in platelet-rich plasma [62]. These effects were at least partly mediated through cyclooxygenase 1 (COX-1) inhibition and thus decreased platelet aggregation, increasing cyclic nucleotide levels, decreasing intracellular and total calcium mobilization and thromboxane B2 (TXB2) levels. In addition, quercetin inhibited the access of AA to the catalytic site of COX-1. Indeed, this resulted in a decrease in inflammation and platelet aggregation. Flavonols were also shown to inhibit platelet function and thrombus formation through effects on early activatory processes, where two quercetin metabolites, isorhamnetin and tamarixetin, interacted with aspirin and enhanced antiplatelet efficacy [63]. Quercetin derivatives in combination with aspirin may be potentially used in prevention and treatment of cardiovascular diseases associated with platelet hyperactivation. However, this clinical effect may be questionable as bioavailability of favonols is low. In vitro anti-platelet activity of phenolic compounds was shown only at very high concentrations that are unlikely to be achieved in vivo [64]. Nevertheless, it should be taken into account that flavonol metabolites formed by colon microbiota may reach significantly high plasma levels and exert a biologically relevant antiplatelet effect [65].

In line with the above investigation, the effect of flavonols on lipid metabolism has been also investigated. Flavonols have been demonstrated to reduce hyperlipidemia and atherosclerotic-lesion formation. The action of flavonoids appears to be multifaceted and dependent on parallel processes. Li et al. determined the effects of *Allium cepa* extract, rich in quercetin and isoquercetin, on hyperlipidemia Sprague Dawley (SD) experiment rat models [66]. *Allium cepa* extract reduced total cholesterol (TC), triglycerides (TG), low-density lipoprotein cholesterol (LDL-c), and malondialdehyde (MDA) and increased high-density lipoprotein cholesterol (HDL-c) in SD rats. Further, the extract promoted the degradation of 3-hydroxy-3-methylglutaryl-coenzyme A reductase (HMGCR) and increased LDL-receptor (LDLR) expression in the liver. Downregulation of HMGCR lowered intracellular cholesterol concentrations, which led to an increase in LDL-receptor expression. This, in turn, increased cellular lipoprotein absorption and cholesterol elimination from the circulation. It is recognized that reverse cholesterol transport (RCT) protects against atherosclerosis by transporting excess cholesterol from peripheral tissues to the liver and small intestine for excretion [67]. Consistent with this, a recent study confirmed that atherosclerosis mice fed a high-fat diet (15% fat and 1.25% cholesterol) and quercetin-supplemented diet (12.5 mg/kg/day) improved RCT [68]. In this study, quercetin intake led to increased cholesterol-accepting ability of plasma and high-density lipoprotein (HDL) and decreased the content of MDA in plasma and oxidized phosphocholine carried by HDL. It is interesting to note that the underlying mechanism of quercetin’s in vivo action is possibly attributed to the increased cholesterol-accepting capacity of HDL; the increased expression levels of proteins related to RCT, such as ATP-binding cassettes (ABC) A1 and G1; and the reduction in oxidation. Similarly, Li. et al. confirmed that quercetin blocked damage of ox-LDL-induced RAW264.7 cells and improved viability, as well as reduced lipid accumulation and senescence by regulation of ABCAl, ABCG1, liver X receptor-α (LXR-α), proprotein convertase subtilisin/kexin type 9 (PCSK9), P53, P21, and P16 expression [69]. These findings help clarify the lipid-lowering properties of quercetin (Table 2).

## 7. Clinical Studies on Flavanol Interventions—An Update from the Last 5 Years

Recently, growing attention has been focused on using natural antioxidant sources in the prevention and treatment of cardiovascular diseases, especially for the cardiovascular-risk population. Food flavonoids have shown capability in the prevention of CVDs. Comprehensive analyses of observational studies showed an inverse association between dietary flavonoid intake and mortality, including CVD-related mortality [70]. A recent study involving 39 prospective cohort studies including 1,501,645 individuals reported that higher consumption of flavonols was consistently associated with a lower risk of coronary heart disease (CHD). In addition, intake of quercetin was shown to be linearly associated with a lower risk of CHD, where the lowest risk was observed for up to 12–14 mg day^−1^ [71].

Preclinical data have established flavonols as chemicals with metabolic regulatory actions that may contribute to preventing or delaying the onset of cardiovascular diseases, but human evidence is still limited. The anti-inflammatory effects of flavonols, their impact on the cardiovascular system in both cardiovascular-risk and cardiovascular-disease populations have been the subject of current randomized controlled human trials (Supplementary Table S1). This section of the review focuses on clinical studies from over the past five years which present the strongest evidence for flavonol intake’s preventive function in the manifestation of cardiovascular diseases.

### 7.1. Flavonols and Endothelial Function

The endothelium is directly involved in peripheral vascular diseases. Endothelial dysfunction (ED) is characterized as an imbalance of numerous endothelium-derived relaxing and constrictor factors, as well as an altered metabolizm of available nitric oxide (NO) [72]. As a result, vascular homeostasis is disrupted, manifesting in a prothrombotic, proinflammatory, and less compliant blood-vessel wall. ED is an early event in the development of hypertension and CVD. Recent findings from randomized clinical studies support the beneficial effect of quercetin on endothelial function [73,74].

Apples are one of the most abundant sources of quercetin. Bondonno et al. investigated the effects of acute and/or chronic Cripps Pink apple-extract intake (12.5 mg vs. 195.3 mg of quercetin daily) on endothelial function in 30 participants at risk of cardiovascular disease [73]. Endothelial function was assessed using flow-mediated dilation (FMD) of the brachial artery. Both the acute and the 4-week intervention by 195.3 mg improved FMD. Similarly, a higher FMD response was observed in the group of patients who acutely supplemented enzymatically modified isoquercitrin [74]. These data suggest that quercetin glycosides found in apples potentially modulated endothelial function through the regulation of vascular tone. These results further show that these substances can enhance NO bioavailability, possibly by stimulating endothelial NO synthase (eNOS) activity. Unexpectedly, no benefit in terms of blood pressure (BP) and arterial stiffness was detected by neither Cripps Pink apple intake nor isoquercetin.

### 7.2. Effects of Flavonols on Lipid Profile

Evidence over the past decades and current research shows a link between dyslipidemia and risk of stroke and atherosclerotic cardiovascular disease (ASCVD). Low-density lipoprotein cholesterol (LDL-c) is a well-known modifiable risk factor for ASCVD and a key target for intervention in both primary and secondary prevention of ASCVD.

The effect of flavonols on lipids and several biochemical parameters associated with metabolic syndrome (MetS) in participants at high cardiovascular risk was widely investigated. A twelve-week dietary supplementation with bread enriched with (−)-epicatechin and quercetin (0.05%) improved total cholesterol, LDL-cholesterol, triglycerides, and fasting plasma glucose in 156 participants who had at least three of the risk factors for MetS [75]. Further, the combination therapy (ezetimibe and nutraceutical compound in which quercetin was found) reduced total cholesterol (TC) and LDL-c levels by 25.9% and 38.7%, respectively, among statin-intolerant hypercholesterolemic subjects [76]. It was found that the lipid-lowering effect of red yeast rice, which was administrated in the therapy, may have modulatory effects on the endogenous synthesis of LDL-c by inhibiting the HMGCR. Moreover, this treatment is recommended as an alternative to lipid-lowering therapy in the case of statin intolerance [77]. A study that analyzed data from 18 randomized controlled trials involving 530 subjects reported that chronic supplementation with flavonol (mostly quercetin) had a beneficial effect on blood lipid levels [78]. In the same study, secondary analyses of subgroups revealed that participants from Asian countries and those with diagnosed cardiovascular diseases or dyslipidemia had the greatest benefit on flavonol intake when compared to healthy subjects with normal baseline values.

Not all studies provide evidence to support beneficial effects on TC and LDL-c concentrations in healthy individuals and patients at high cardiovascular risk. A recent intervention trial found that regular lemon-balm-extract intake, rich in flavonols, for 12 weeks did not improve TC and LDL-c parameters in type 2 diabetic patients [79]. Nevertheless, there was a significant change in HDL-c levels. Apart from a significant reduction in triglicerydies at doses above 500 mg/day, quercetin supplementation had no positive effect on plasma lipids according to a meta-analysis by Sahebkar, which included 221 subjects from five randomized controlled trials [80]. Similarly, a recent meta-analysis of 18 randomized controlled studies with a total of 896 participants reported that quercetin administration for 8 weeks or longer had no significant effect on TC and LDL-c levels. However, levels of HDL-c and TG were favorably changed [81]. Taking into account that epidemiological evidence indicates that increased HDL-C concentrations correlate inversely with CVD risk, these findings point to a potentially important clinical benefit of daily flavonol intake, especially quercetin [82].

### 7.3. Effects of Flavonols on Blood Pressure

Hypertension is the leading cause of cardiovascular events such as myocardial infarction, stroke, and heart failure. Reductions in BP through antihypertensive treatment lower the risk of all major cardiovascular events.

The latest research provides evidence for the beneficial effects of flavonols, mostly quercetin on BP, and supports the use of quercetin as a combination therapy in patients with hypertension and those at cardiovascular risk. A recent long-term intervention trial showed that daily quercetin supplementation (1000 mg 2 times per day for 6 months, then 500 mg 2 times per day for a subsequent 6 months) improved BP without increasing the dose or adding new antihypertensive drugs in patients with gout in combination with essential hypertension [83]. One year of intervention led to a 5.5% and 3.6% reduction in systolic BP and diastolic BP, respectively. Dietary supplementation of quercetin reduced levels of uric acid by 33.7% and normalized renal function. Further, administration of lemon-balm extract (700 mg hydroalcoholic extract of M. officinalis/daily per 12 weeks) significantly reduced systolic BP in diabetes patients [79]. However, no beneficial effects on BP were observed in patients after myocardial infarction (MI) [84]. In line with the clinical studies mentioned above, the antihypertensive effects of flavonols were also reported in recent meta-analyses [81,85]. It must be highlighted that these beneficial effects depend on the formulation and dosage of quercetin, as well as the duration of treatment.

### 7.4. Effects of Flavonols on Other Parameters Related to Cardiovascular Health

Current research shows a link between changes in inflammatory profile and the risk of several chronic conditions, including metabolic syndrome, type 2 diabetes mellitus, and CVDs [86,87,88]. Interestingly, the high-flavonol diet was related to a decrease in urinary isoprostanes, which is a well-known marker of oxidative stress [89]. In a recent clinical study, flavonol supplementation appeared to ameliorate inflammatory factors in postmyocardial-infarction patients, with either slight or no effects on proinflammatory markers and antioxidant parameters among overweight and obese adults with hypertension and metabolically healthy men and women [84,90,91,92]. This disparity between results could be due to inflammatory factors being in the normal range at the baseline, low supplementation dosage, a small number of participants, duration, and plasma-flavonol concentration.

Flavonols’ effects on the incidence of venous-system disease, advanced glycation end products (AGEs), and biomarkers of heart health risk have also been reported in recent clinical studies [89,93,94,95]. Blood biomarkers of heart health, such as homocysteine (Hcy), high-sensitivity C-reactive protein (hs-CRP), oxidized LDL (ox-LDL), gamma-glutamyl transferase (GGT), uric acid, and blood lipid profile were investigated among the Russian population under a multivitamin, multimineral, and phytonutrient (VMP) supplement which contained quercetin. An eight-week intervention reduced serum Hcy and GGT [94]. Flavonols were also shown to influence plasma concentrations of methylglyoxal (MGO). MGO is a crucial component in the rapid formation of advanced glycation end products (AGEs). MGO and AGEs have been linked to diabetes and its consequences. An intervention trial showed that daily quercetin intake (160 mg) for 4 weeks reduced MGO by 10.6% from baseline values in healthy (pre)hypertensive participants [93]. Given these findings, it is tempting to speculate that quercetin’s MGO-scavenging effect could lead to a new treatment strategy for diseases in which MGO plays a key role.

Flavonol treatment was also investigated in extremity-immobilization patients. Immobilization can be a cause of venous stasis and deep-venous thrombosis (DVT), which included vessel-wall damage, stasis or low flow, and hypercoagulability. O-(b-Hydroxyethyl)-rutosides (Oxerutin) are a standardized combination of the semisynthetic flavonoids created by replacing the hydroxyl groups with hydroxyethyl groups in the naturally occurring flavonol rutin. Yildiz et al. showed that 6–8-week Oxerutin intake reduced the incidence of superficial venous reflux in the distal crural veins in cast immobilization for lower-limb-fracture patients [95].

## 8. Summary

Cardiovascular diseases are the group of disorders that continue to be a leading cause of morbidity and mortality around the world. Understanding the significance of a natural food supplement as a novel avenue of investigation for the implementation of novel cardioprotective strategies and the management of current therapies would contribute to the prevention of CVDs. The assessment and treatment of various modifiable CVD risk factors, such as elevated cholesterol levels, specially LDL-c, high blood pressure, and high blood glucose, is required for optimum management of individuals with or at risk for CVDs.

The present narrative review provides a synthesis of results from randomized controlled trials on the cardioprotective effects of flavonols, especially quercetin. At least 19 clinical data have been conducted in the last five years to examine the relationship between flavonol supplementation and cardiovascular disease markers. The major result was that intake of flavonols—mainly quercetin—may modulate endothelial function, has lowering effects on plasma lipids, and may ameliorate blood hypertension in different groups of patients. However, the current analyses detected inconsistencies between the results of RCTs and meta-analyses. The first limitation of the presented clinical data is relatively heterogeneous populations (e.g., healthy participants as well as those with MetS, obesity, hypertension, or patients after myocardial infarction) in terms of the inherent risk of CVD. Moreover, an unbalanced gender ratio was observed. More limitations include a short duration of observation, limited sample sizes, and a wide range of flavonol-supplement types and dosages. Finally, the combined effects of flavonols and other phytochemicals used in some of the included trials may limit the current findings.

Regarding flavonols’ effects on human cardiovascular health, future research on flavonols should focus on the numerous food sources of flavonols, the effective flavonol dosage, and the optimal frequency and duration of flavonol intake. The majority of presented trials were short-term or observed acute effects of flavonol intake. Thus, there is a need for more research to examine the impact of long-term flavonol intake and to identify any potential side effects of their use. It is important to notice that none of the included studies found that investigated substances caused serious adverse events. Finally, future research on flavonols should concentrate on interindividual variability in flavonoid metabolism as it relates to the gut microbiome and nutrient interactions.

In conclusion, the findings of this review provide evidence that a flavonol-rich diet can help protect against the rising trends of the world’s leading cause of death. The present review would help doctors and other healthcare workers understand the role of both supplements and natural sources of flavonols, especially quercetin in cardiovascular-disease prevention and management.

## Figures and Tables

**Table 1:** Content of flavonols in selected foodstuffs (mg per 100 g of foodstuff), based on [20].

	Flavonols (mg/100 g), Edible Portion				
Product	Quercetin	Kaempferol	Myricetin	Isorhamnetin	Total
Fresh capers	233.84	259.19	nd	nd	493.03
Dried parsley (*Petroselinum crispum*)	0.0	0.0	nd	331.24	331.24
Saffron (*Crocus sativus*)	nd	205.48	nd	nd	205.48
Dill weed (*Anethum graveolens*)	55.15	13.33	0.70	43.50	112.68
Elderberry juice concentrate	108.16	nd	nd	nd	108.16
Sorrel (*Rumex* spp.)	86.20	10.30	5.70	0.00	102.20
Kale (*Brassica oleracea* (*Acephala Group*))	22.58	46.80	0.00	23.60	92.98
Fennel, leaves	48.80	6.50	19.80	9.30	84.40
Rocket lettuce (*Diplotaxis tenuifolia*)	66.19	1.78	nd	0.78	68.75
Coriander (*Coriandrum sativum*)	52.90	0.00	nd	0.00	52.90
Arugula (*Eruca sativa*)	7.92	34.89	nd	4.30	47.11
Red onions	39.21	0.70	2.16	4.58	46.65
Carob flour (*Ceratonia siliqua*)	38.78	0.44	6.73	nd	45.95
Elderberries (*Sambucus* spp.)	26.77	0.58	nd	5.42	32.77
Ginger (*Zingiber zerumbet*)	0.00	33.60	0.00	0.00	33.60
Goji berries	nd	6.20	11.40	13.60	31.20
Chia seeds	18.42	12.30	nd	nd	30.72
Fresh Cranberries (*Vaccinium macrocarpon*)	16.64	0.09	7.63	nd	24.36
Chard (*Beta vulgaris* subsp. *Vulagaris*)	7.50	9.20	2.20	nd	18.90
Chokeberry	18.53	0.34	0.00	nd	18.87
Dried and sweetened cranberries	12.79	0.01	5.67	nd	18.47
Mizuna (Japanese mustard)	8.55	6.03	nd	3.84	18.42
Chives	0.00	17.11	0.00	0.00	17.11
Buckwheat (*Fagopyrum esculentum Moench*)	15.38	nd	nd	nd	15.38
Cooked asparagus	15.16	nd	nd	nd	15.16
Plums, black diamond	12.45	0.01	0.01	0.00	12.47
Blackcurrants (*Ribes nigrum*)	4.45	0.71	6.18	0.12	11.46
Spinach (*Spinacia oleracea*)	3.97	6.38	0.35	nd	10.70
Blueberries (*Vaccinium* spp.)	7.67	1.66	1.30	nd	10.63
Endive (*Cichorium endivia*)	0.00	10.10	0.00	nd	10.10
Chicory	6.49	2.45	0.0	nd	8.94
Fresh figs	5.47	0.00	0.00	nd	5.47
Cooked Brussel sprouts	4.33	0.91	nd	nd	5.24
Apples, Gala, raw	3.80	0.00	0.00	nd	3.80

**Table 2:** In vivo and in vitro research update (studies published in 2021) on flavonols and their cardioprotective effects in pathological conditions.

Disorder/Substances	In Vitro or In Vivo Model		Mode of Action	References
Atrial Fibrillation				
Quercetin	Human isoprenaline (ISO)-induced atrial fibrillation tissues, ISO-induced rats		Regulating miRNA expression, inhibiting the proliferation, myofibroblast differentiation, and collagen deposition in ISO-treated rat cardiac fibroblasts (RCFs)	Wang et al. [40]
Quercetin	Atrial fibrillation model cells and aged-rat myocardial tissues	↑	Autophagy via regulating miRNA-223-3p/FOXO3	Hu et al. [41]
Myocardial Inflammation and Inflammatory Markers				
*Ulva fasciata* methanolic extract (polyphenolics that contain quercetin and rutin)	Hyperthyroidism-associated heart inflammation albino rat model	↓ ↓ ↓	TNF-α, MPO, and CRP TG, TC Cardiac biomarkers CK-MB, LDH, and troponin ROS-scavenging potential	Ibrahim et al. [42]
*Sorbus aucuparia* L. fruits extract (rowanberry) (quercetin contained)	Human blood buffy coats	↑	Inhibiting the formation of AGEs Protecting the plasma proteins and lipids against nitration and oxidation The nonenzymatic antioxidant capacity of plasma Neutralizing multiple oxidants generated in vivo	Rutkowska et al. [43]
Quercetin and lycopene	ISO-induced cardiac injury toxicity in Sprague Dawley (SD) rats	↓ ↓	Myocardial damage Oxidative-stress markers Activatingenzymic antioxidant defense gene-expression pathways	Chen et al. [44]
Quercetin	Mouse cardiomyocytes under inflammatory conditions		Inhibiting the inflammatory response and oxidative-stress injury Inhibiting myocardial fibrosis	Chang et al. [53]
Quercetin	Hypoxia or reoxygenation human cardiomyocytes		Inhibiting oxidative-stress damage Regulating mitophagy and endoplasmic-reticulum stress via SIRT1/TMBIM6	Chang et al. [52]
Myocardial Ischemia-Reperfusion Injury				
Quercetin-loaded mesoporous silica nanoparticles (Q-MSNs)	Myocardial ischemia-reperfusion injury, rats	↓	Enhancing the activation of the JAK2/STAT3 pathway Inhibiting cell apoptosis and oxidative stress Myocardial infarction size	Liu et al. [45]
Quercetin	Ischemia/reperfusion-induced rat model	↓	1β (IL-1β), IL-6 and TNF-α Inhibiting mitoKATP channels Blocking NO system	Liu et al. [54]
Hypercholesterolemia				
*Allium cepa* extract	Hyperlipidemia Sprague Dawley rats	↓ ↑ ↓	TC, TG, LDL-c, MDA HDL-c LDL-receptor expression in liver Degradation of HMGCR	Li et al. [66]
Arterial Vascular Calcification				
Mg combined with polyphenols: quercetin, curcumin, resveratrol	Rat vascular smooth-muscle cells		Synergistic effect in inhibiting vascular calcification Inhibition of calcium deposition	Mehansho et al. [46]
Cardiomyocyte Hypertrophy				
Quercetin and rutin	Ang II-induced cardiomyocyte hypertrophy		Rutin and quercetin had similarly prevented Ang II-induced cardiomyocyte hypertrophy by blunting the ROS/NO axis	Siti et al. [47]
Endothelial Dysfunction				
Sugar apple and unripe papaya, which contained gallic acid and quercetin	Human endothelial cells		Promotion of endothelial function Inducing cell migration and vascular capillary-like tube formation	Wattanapitaya-kul et al. [48]
Cardioprotection				
Quercetin with sitagliptin (anti-diabetic medication)	Doxorubicin (DOX)-induced cardiotoxicity, Wistar rats	↓ ↑	Troponin, LDH, CKP, CRP, TC, LDL-c, TG, atherogenic index of plasma TAOC	Aziz et al. [49]
Quercetin	High-fat diets, (HFD) mice	↓	Normalization of heart weight and TG Cardiac fibrosis, cardiomyocyte hypertrophy, oxidative stress, intramyocardial fat deposition, and vascular rarefaction	Yu et al. [50]

↑—increase, ↓—decrease, Ang II—angiotensin II, AGEs—advanced glycation end products, CK-MB—creatine kinase-MB isoenzyme, CKP—creatine phosphokinase, CRP—C-reactive protein, FOXO3—Forkhead Box O3, HDL-c—high-density lipoprotein cholesterol, HMGCR—3-hydroxy-3-methylglutaryl-coenzyme A reductase, IL—interleukin, JAK2—janus kinase 2, LDL-c—low-density lipoprotein cholesterol, LDH—lactate dehydrogenase, MDA—malondialdehyde, miRNA—microRNA, mitoKATP—mitochondrial ATP-sensitive potassium channel, MPO—metalloproteinase, NO—nitric oxide, ROS—reactive oxygen species, SIRT1—silent information regulator protein 1, STAT3—signal transduction and activator of transcription 3, TAOC—total antioxidant capacity, TC—total cholesterol, TG—triglycerides, TMBIM6—transmembrane BAX inhibitor-1 motif-containing 6, TNF-α—tumor necrosis factor-alpha.

## Data Availability

Not applicable.

## Supplementary Materials

The following supporting information can be downloaded at: https://www.mdpi.com/article/10.3390/nu14071439/s1, Table S1: The summary of (A) clinical trials in particular disorders and (B) meta-analyses.
