# Supplementary material for: Targeting Cardiovascular Diseases by Flavonols: An Update
Source: Nutrients. 2022 Mar 30;14(7):1439. doi: 10.3390/nu14071439 (PMC9003055; doi:10.3390/nu14071439)
Supplement: Supplementary file 1 [file nutrients-14-01439-s001.zip › nutrients-1631354-supplementary.pdf]

Supplementary materials

Targeting Cardiovascular Diseases by Flavonols: An Update

Aleksandra Kozłowska [1] and Dorota Szostak-Węgierek [2,*]

Table S1. The summary of (A) clinical trials in particular disorders and (B) meta-analyses.

Study (year)	Study Design and Comparator (if applicable)	Number of participants	Drug/Substance	Dosage [mg/d]	Condition	Treatment/ use duration	Endpoints/Measures	Outcomes	Limitations
**A. Clinical trials in particular disorders**									
**ENDOTHELIAL FUNCTION**									
Bondonno (2018) [73]	a randomized, controlled cross-over trial	30	low flavonoid apple, LFA vs high flavonoid apple, HFA	195.3 mg of quercetin daily , Cripps Pink apple extract	individuals at risk for cardiovascular diseases (CVDs)	Acute effects or 4 weeks	• endothelial function assessed using flow-mediated dilation (FMD) of the brachial artery<br>• blood pressure (BP)<br>• arterial stiffness	• significant increase in FMD acutely (0.8%) and after 4 weeks chronic intake (0.5%), and in plasma flavonoid metabolites after HFA	it was not feasible for participants to be blinded to the treatment they were receiving
Bondonno (2020) [74]	a randomized, controlled, cross-over trial	25 aged between 50 and 70 years	EMIQ®, Enzymatically modified isoquercitrin,	4.89 mg EMIQ® (2 mg aglycone equivalent)/kg body weight vs placebo	participants with at least one CVDs risk factor	Acute effects	• endothelial function<br>• BP<br>• arterial stiffness<br>• cognitive function<br>• oxidative stress<br>• markers of nitric oxide (NO) production	• higher FMD response compared with the placebo<br>• plasma concentrations of quercetin metabolites were significantly higher after EMIQ® treatment	small sample size
Brüll (2017a) [91]	a randomized, double-blind, placebo-controlled, crossover trial	22	quercetin from onion skin extract	54 mg of quercetin vs placebo	overweight and obese adults with hypertension	Acute effects	• metabolic and vascular responses<br>• BP<br>• reactive hyperemia index (RHI)<br>• high-sensitive C-reactive protein (hs-CRP)<br>• soluble endothelial-derived adhesion molecules<br>• parameters of lipid and glucose metabolism<br>• markers of antioxidant status	• Postprandial metabolic responses induced by the challenge, such as lipemia and insulinemia, were not attenuated by the concomitant ingestion of quercetin<br>• no acute effects of quercetin on markers of endothelial function	time points at which endpoints were measured may not be representative of the entire postprandial period
**CARDIOVASCULAR RISK FACTORS**									
Asadi (2019) [79]	a randomized, double-blind, trial	62	*Melissa officinalis* L. (lemon balm) extract	700-mg hydroalcoholic extract of M. *officinalis*/daily	patients with T2DM	12 weeks	• cardiovascular risk factors<br>• glycemic control	• reduction in fasting blood glucose level, hemoglobin A1c	small sample size, using lower dosage

					(type 2 diabetes mellitus)			(HbA1c) , systolic BP and triglycerides (TG), hs-CRP level<br>• increase in high-density lipoprotein (HDL-c) levels and mean change of paraoxonase-1 (PON1)<br>• insulin, homeostasis model assessment-insulin resistance (HOMA-IR), and pancreatic β-cell function were significantly decreased	of M. *officinalis* supplementation
Van den Eynde (2018) [93]	a randomized, double-blind, placebo-controlled, crossover trial	37	quercetin 3-glucoside, epicatechin<br>or placebo	160 mg/d<br>100mg/d	healthy (pre)hypertensive men and women	4 weeks	• methylglyoxal (MGO)<br>• advanced glycation end products (AGEs)	• quercetin treatment reduced MGO by 10.6% from baseline values	relatively healthy individuals with a short intervention period
Isakov (2018) [94]	a randomized, double-blind, placebo -controlled trial	120	multivitamin, multimineral and phytonutrient supplement which contained quercetin	no data	population with low fruit and vegetable intake	8 weeks	• biomarkers of heart health risk<br>• nutritional status (blood levels of β-carotene, α-tocopherol, vitamin C, B6, B12, red blood cell, folate, zinc, selenium, and quercetin)	• improved the nutritional status<br>• reduced biomarkers of heart health risk: serum homocysteine (Hcy), serum gamma-glutamyl transferase (GGT)<br>• no significant effects on the lipid profile	unbalanced gender ratio<br>effects of many substances
Burak (2019) [92]	a randomized, double-blind, placebo-controlled, crossover trial	67	Alpha-linolenic acid (ALA) and quercetin<br>Vs.<br>ALA+ placebo	3.6 g/d<br>190 mg/d	metabolically healthy men and women	8 weeks	• effects on BP and lipid and glucose metabolism<br>• biomarkers of inflammation, oxidative stress, and antioxidant status	• no effects on BP<br>• no effect on HDL-c, apolipoprotein A1, glucose, uric acid, oxidized low-density lipoprotein (*oxy*LDL), CRP<br>• both interventions decreased total cholesterol (TC), low-density lipoprotein cholesterol (LDL-c), non-high-density lipoprotein cholesterol, and apolipoprotein B	the present study population was metabolically healthy<br>combined effects of a macronutrient ALA and a phytochemical (quercetin)

Kondratiuk (2018) [83]	a randomized, double-blinded, trial	84	quercetin	1000 mg 2 times per day for 6 months, then 500 mg 2 times per day for subsequent 6 months	men with gout and essential hypertension	12 months	• echocardiographic parameters of the left ventricular diastolic function	• no evidence was seen for an additive or synergistic effect of ALA plus quercetin on markers of cardiovascular disease risk.<br>• improve echocardiographic parameter of diastolic function left ventricular, purine metabolism, renal function<br>• reduction of systolic BP by 5,5% and diastolic BP by 3,6%	study conducted only among men
Leyva-Soto (2021) [75]	a randomized placebo-controlled trial	156	enriched bread with epicatechin and quercetin	bread with 0.05% of a 1:1 mixture of (−)-epicatechin and quercetin	adults who have at least 3 of the risk factors for Metabolic Syndrome (MetS)	12 weeks	• biochemical parameters related to metabolic syndrome<br>• genotoxicity in buccal epithelium cells	• decreased TC, LDL-c, triglycerides (TG), and fasting plasma glucose<br>• nuclear abnormalities in buccal epithelium cells also decreased	flavonoid content significantly decreased during storage
Vetrani (2018) [89]	a randomized controlled parallel-group trial	78	intake of polyphenol (PP) subclasses with different types of diet	low PP diet: ~365 mg/ day vs. high PP diet: ~2903 mg/day	participants at high cardiovascular risk	8 weeks	• cardiometabolic risk factors	• The high flavonol intake was related to decrease in urinary isoprostanes	the effects of a whole diet rich in polyphenols
**POST-MYOCARDIAL INFRACTION PATIENTS**									
Dehghani (2021) [84]	a randomized, double- blind, placebo-controlled, trial	76	quercetin supplementation	500 mg/day	patients following myocardial infarction (MI)	8 weeks	• inflammatory factors,<br>• total antioxidant capacity (TAC)<br>• quality of life (QOL)	• increased serum total antioxidant capacity (TAC)<br>• decreased in TNF-α levels<br>• improved insecurity dimension of QOL<br>• no effects on interleukin 6 (IL-6), hs-CRP, BP	lack of measurement of quercetin metabolites in plasma or urine of patients
**SYSTEMIC AND ADIPOSE TISSUE INFLAMMATION**									
Brüll (2017b) [90]	a randomized double-blind, placebo-controlled crossover trial	68	quercetin from onion skin extract	162 mg/d	overweight and obese adults with pre- and stage 1 hypertension	6 weeks	• biomarkers of inflammation, leptin, adiponectin, glucose and insulin levels	• no effects on serum concentrations of leptin and adiponectin, Homeostasis model assessment-	only measured total serum adiponectin and not the potentially more biologically relevant high

							• safety of daily quercetin supplementation	adiponectin (HOMA-AD) or the ratios of leptin/adiponectin and adiponectin/leptin. • No effects on hsCRP and plasma tumor necrosis factor alpha (TNF-α)	molecular weight adiponectin concentration
**STATIN-INTOLERANT HYPERCHOLESTEROLEMIC PATIENTS**									
Mazza (2020) [76]	an open-label randomized single-center study	96	Colenorm Cardio	3 mg of monacolin-K, 100mg of quercetin, 50mg of berberine hydrochloride, 20mg of t-resveratrol, 50 mcg of chromium, and 5.25mg of black pepper	statin-intolerant hypertensive and hypercholesterolemic patients with moderate-to-high CV risk	12 weeks	• lipid profile	• decrease in the TC (-25.9%) and LDL-c levels (-38.7%) • No changes in TG and HDL-c	single center combined effects of many substances
**VENOUS DISEASE**									
Yildiz (2017) [95]	A randomized controlled trial	60	Venoruton	500 mg O-(b-Hydroxyethyl)-rutosides, twice a day	patients with calf muscle pump dysfunction	until removal of the cast, 6–8 weeks	• incidence of venous system disease	• reducing the incidence of reflux in the below-knee superficial veins	lack of histopathologic examination of vascular structures
**B. META-ANALYSES**									
**Study (year)**	**Study Design and Comparator**	**Number of trials (subjects)**	**Drug/Substance**	**Dosage [mg/d]**	**Condition**	**Treatment/ use duration**	**Endpoints/Measures**	**Outcomes**	**Limitations**
Micek (2021) [71]	Meta-analysis of prospective cohort studies	39 studies (1 501 645 individuals)	dietary flavonoids subclasses	the highest versus the lowest category of flavonoids subclasses intake	subject from United States, Europe, Asia and Australia	n/a	• dietary intake of total, subclasses and individual flavonoids and risk of cardiovascular disease	• intake of quercetin is linearly associated with lower risk of coronary heart diseases (CHD), the lowest risk was observed for up to 12–14 mg day^{-1} • increasing intake of flavonols is inversely associated with CHD	result based on risk estimates extracted from observational population studies, which do not allow to fully assess a cause-effect relation
Huang (2020) [81]	Meta-analysis of randomized controlled trials	17 trials (896 subjects)	quercetin	the dosage of quercetin ranged from 30 mg/d to 1000 mg/d	healthy patients, patients with rheumatoid arthritis, hypertension, prehypertension, prehyperuricemia,	2-12 weeks	• effect on plasma lipid concentrations • effects on blood pressure and glucose concentrations • clinical safety	• decreased BP supplementation for 8 weeks or more showed significantly increased levels of HDL-c and decreased levels of TG	relatively heterogenous populations

					polycystic ovary syndrome, T2DM, obesity, overweight				
Tamtaji (2019) [85]	Meta-analysis of ran-domized con-trolled trials	9 trials	quercetin	n/a	patients with MetS and related disorders.	n/a	• effect on BP and endothelial function	• reduced systolic BP<br>• no effects on diastolic BP, vascular cell adhesion molecule 1 (VCAM-1) and (intercellular adhesion molecule 1) ICAM-1	n/a
Menezes (2017) [78]	Meta-analysis of ran-domized controlled trials	18 trials (530 subjects)	pure flavonol supplements or enriched mixtures of flavonols	16 -1200 mg/day of flavonol	healthy and unhealthy participants (hypertension, metabolic diseases)	14-90 days	• cardiometabolic biomarkers: BP, TC, HDL-c, LDL-c, TG, glucose, FMD, HbA1c, HOMA-IR	• long-term supplementation with flavonols (mostly quercetin) has a beneficial effect on blood lipid levels<br>• reduction in TG and LDL-c levels<br>• increase of HDL-c<br>• reduction for diastolic BP and systolic BP<br>• reduction in fasting glucose levels	relatively heterogenous pop-ulations
Sahebkar (2017) [80]	Meta-analysis of ran-domized controlled trials	5 trials (221 subjects)	quercetin	30-730 mg/day	Healthy and unhealthy participants (metabolic disease)	2-10 weeks	• lipid profile: TC, LDL-c, HDL-c, TG	• reduction of TG at doses above 500 mg/day<br>• no clinically relevant effect of quercetin on other plasma lipids	small population sizes of individual studies<br>only assessed RCTs with pure quercetin aglycone and quercetin dehydrate

AGEs— advanced glycation end products, ALA— alpha-linolenic acid, BP— blood pressure, CHD— coronary heart diseases, FMD— flow-mediated dilation, GGT— gamma-glutamyl transferase, Hcy— homocysteine, HbA1c— hemoglobin A1c, HDL-c— high-density lipoprotein, HOMA-AD— homeostasis model assessment-adiponectin, HOMA-IR— homeostasis model assessment-insulin resistance, hs-CRP— high-sensitive C-reactive protein, ICAM-1— intercellular adhesion molecule 1, IL-6— interleukin 6, MetS— metabolic syndrome, MGO— methylglyoxal, MI— myocardial infarction, n/a—not available or not applicable, NO— nitric oxide, *oxy*LDL— oxidized low-density lipoprotein, PON1— paraoxonase-1, QOL— quality of life, RHI— reactive hyperemia index, T2DM— type 2 diabetes mellitus, TAC— total antioxidant capacity, TC— total cholesterol, TG— triglycerides, TNF-α— tumor necrosis factor alpha, VCAM-1— vascular cell adhesion molecule 1, .
